# The European Nucleotide Archive in 2019

**DOI:** 10.1093/nar/gkz1063

**Published:** 2019-11-13

**Authors:** Clara Amid, Blaise T F Alako, Vishnukumar Balavenkataraman Kadhirvelu, Tony Burdett, Josephine Burgin, Jun Fan, Peter W Harrison, Sam Holt, Abdulrahman Hussein, Eugene Ivanov, Suran Jayathilaka, Simon Kay, Thomas Keane, Rasko Leinonen, Xin Liu, Josue Martinez-Villacorta, Annalisa Milano, Amir Pakseresht, Nadim Rahman, Jeena Rajan, Kethi Reddy, Edward Richards, Dmitriy Smirnov, Alexey Sokolov, Senthilnathan Vijayaraja, Guy Cochrane

**Affiliations:** European Molecular Biology Laboratory, European Bioinformatics Institute, Wellcome Genome Campus, Hinxton, Cambridge CB10 1SD, UK

## Abstract

The European Nucleotide Archive (ENA, https://www.ebi.ac.uk/ena) at the European Molecular Biology Laboratory’s European Bioinformatics Institute provides open and freely available data deposition and access services across the spectrum of nucleotide sequence data types. Making the world’s public sequencing datasets available to the scientific community, the ENA represents a globally comprehensive nucleotide sequence resource. Here, we outline ENA services and content in 2019 and provide an insight into selected key areas of development in this period.

## INTRODUCTION

For the last 37 years, since the European Molecular Biology Laboratory (EMBL) launched the first EMBL nucleotide sequence database library, major advances in sequencing and archiving technologies have led to a broad range of nucleotide sequences that build the content of today’s European Nucleotide Archive (ENA). The spectrum extends from raw reads to assembled and annotated sequences and related data types. Having a broad user profile, the ENA offers both general support for the world’s sequence data operations and specific thematic collaborative data coordination (see the ‘Data Coordination Services’ section). As a founding partner in the International Nucleotide Sequence Database Collaboration (INSDC, www.insdc.org) (1), ENA represents a globally comprehensive nucleotide data resource, contributes towards data standards and moves forward with technological advances in sequencing. As an ELIXIR (https://elixir-europe.org/) Core Data Resource (https://elixir-europe.org/platforms/data/core-data-resources), the ENA has a mission to contribute towards the FAIR guiding principles for data management and discovery (2). This mission is achieved by various means: public data stored in the ENA are ‘findable’ through various search tools covering both programmatic and interactive options to provide maximum flexibility for ENA users. Public data are also ‘accessible’ both directly through the ENA and globally though the INSDC exchange. ‘Interoperability’ is provided through structured data and metadata formats that are validated at the time of reporting. Finally, ‘reusability’ is supported through promotion of data sharing and clear terms of use (https://www.ebi.ac.uk/about/terms-of-use). An important tool in assisting users with FAIR compliance for their datasets, the ENA reaches high levels of compliance for most of its content and strives to improve its services further for greater compliance and user value.

Throughout 2019, we have continued to provide services to our user base and have developed in selected key areas. In this article, we focus on data submissions, the introduction of new data classes and metadata standards, the ENA’s expanded data coordination portfolio linked with these services and last, but not least, we highlight the new ENA Browser as one of the year’s significant new offerings.

## ENA CONTENT AND DEPOSITION SERVICES

In 2019, we have continued to operate our open services for user support, submissions, archiving, presentation and discovery of nucleotide sequence data. Table 1 lists ENA services and their entry points.

**Table 1.** ENA services and the respective entry points

Services	Service entry points	Purpose of service	Link to service
User support	Support form	Contact and feedback to Helpdesk	https://www.ebi.ac.uk/ena/browser/support
	Support documentation	Submission, update and discovery guidelines and FAQs	https://ena-docs.readthedocs.io/en/latest/
Data submission	Submission tools	Provision of various submission tools	https://www.ebi.ac.uk/ena/browser/submit
Data access	ENA Browser	Provision of various search tools	https://www.ebi.ac.uk/ena/browser/search

**Figure 1.**
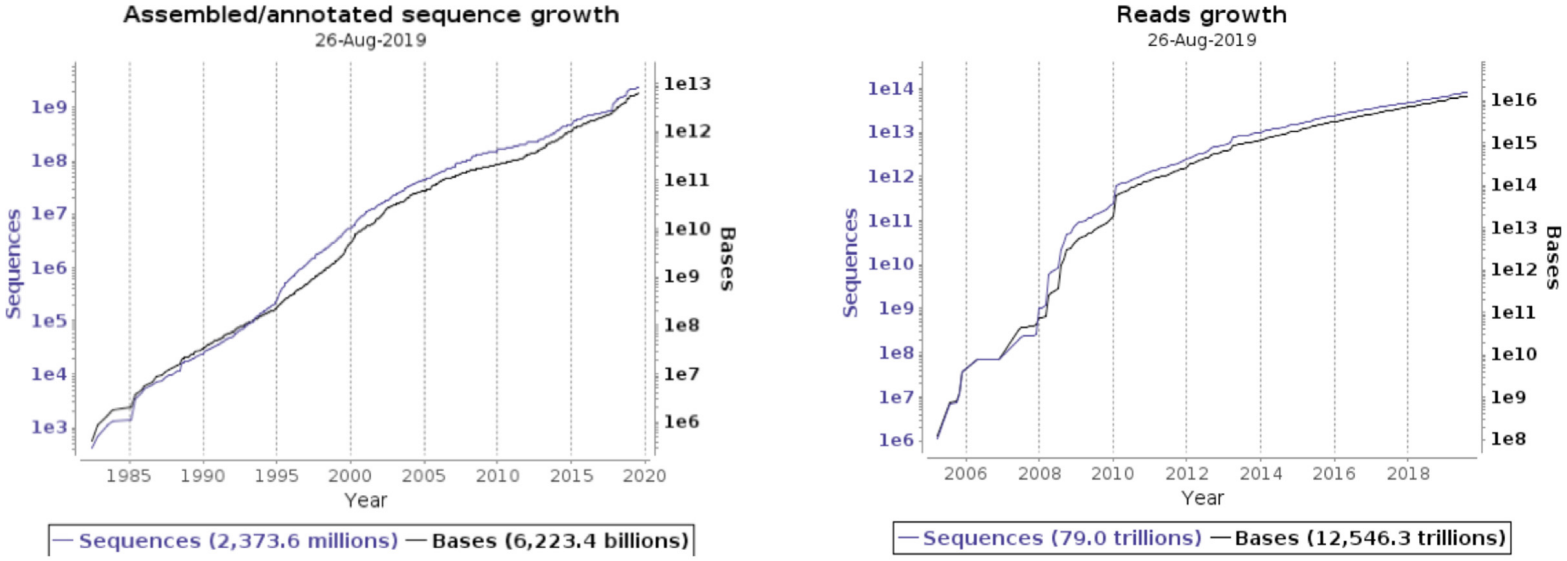
Data growth of total content, by assembled/annotated sequences and reads.

During the past year, we have supported substantial data growth and delivered major new components. The Webin framework has continued to provide for ENA’s deposition services, with a few recently applied changes towards simplification and streamlining on both the submitter and ENA Helpdesk support sides. While metadata registration services (studies and samples) are still supported by interactive and programmatic Webin, a Command Line Interface (Webin-CLI) introduced in 2018 (3) has become ENA’s primary submission tool for genomes and transcriptomes, but also supporting reads and annotated sequences (https://ena-docs.readthedocs.io/en/latest/submit/general-guide.html). Webin-CLI is provided in the form of a standalone executable JAR file, which can be downloaded from https://github.com/enasequence/webin-cli/releases and run from a UNIX terminal or Windows command prompt, and has the major advantage of supporting a pre-submission validation functionality. The Webin submission interfaces have provided support to several thousand active data submitters from numerous countries over the last year, covering 419 490 direct submissions to the ENA in 12 months, comprising 5700 studies, around 620 000 samples, 493 000 runs and 197 000 (meta)genome assemblies. Figure 1 shows the data growth of total content in ENA, which includes the extensive data exchange with the INSDC partners.

## SELECTED DEVELOPMENTS IN 2019

### New data types

The ENA has continued to adapt rapidly and in an agile way to emerging community requirements. We have added support for new sequencing platforms and experiment types and built services around diverse new analysis data types, including 10x reads (https://www.10xgenomics.com/) and metagenome assemblies. In recent years, ENA has focused its extensibility into its analysis objects. Examples of new analysis types in the last year include new assembly classes and taxonomic reference data detailed below.

### Assemblies

**Figure 2.**
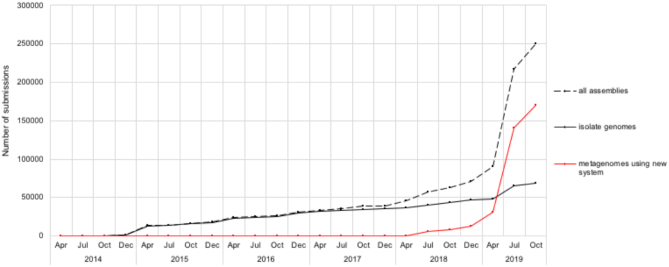
Cumulative number of assemblies submitted to ENA classed by type.

In response to a growing metagenomics world, ENA introduced new analysis classes for primary metagenome, binned metagenome, metagenome-assembled genome (MAG) and single-cell amplified genome, and has implemented accompanying community metadata standards (4). The new analysis types offer an opportunity to explore a new generation of assembly submission and storage. This is achieved by the separation of high-volume primary and binned metagenomes that are difficult to handle in traditional flat files from other, for example, MAGs or isolate genome assemblies. The new and separate analysis types also allow better indexing of the different data groups enabling an improved search and presentation of the data. To ease support for our (meta)genomic assembly submitters, we have added comprehensive documentation describing the new assembly model (https://ena-docs.readthedocs.io/en/latest/submit/assembly.html; https://ena-docs.readthedocs.io/en/latest/submit/assembly/metagenome.html). Figure 2 shows the cumulative number of assemblies submitted to ENA by type.

### Taxonomic reference datasets

In environmental sequencing (e.g. metabarcoding), there is a need to map unknown sequences to taxonomically classified and curated reference sequences. Sets of these reference sequences are typically derived from ENA sequences that are cleaned up (e.g. trimmed and contamination-screened) and mapped to improve and correct taxonomy. Reference datasets are produced by groups (e.g. SILVA: https://www.arb-silva.de/; ITSoneDB: http://itsonedb.cloud.ba.infn.it/; UNITE: https://unite.ut.ee/) (5-7) that consume ENA, add value through such curation processes and make their data available to tool and service providers. With this new analysis class, we support this community data flow.

### Standards

The ENA has continued working with communities to develop and deploy data standards, with a main focus on metagenomics this year. In collaboration with the Genomic Standards Consortium (https://press3.mcs.anl.gov/gensc/), we have deployed three new sample checklists that can be found under the ‘Environmental checklists’ group in Webin:

- MIMAGs, for metagenome-assembled genomes (4);
- MISAGs, for environmental single-cell amplified genomes (4);
- MIUVIG, for environmental/uncultivated virus genomes (8).

In addition to the above, we have also deployed ENA binned metagenome sample checklists to support all levels of assemblies derived from a biome, with corresponding documentation (https://ena-docs.readthedocs.io/en/latest/faq/metagenomes.html; https://ena-docs.readthedocs.io/en/latest/submit/assembly/metagenome.html).

With these, the ENA offers 21 environmental sample checklists that can be selected from based on the biome the sequenced sample is derived from. Furthermore, there are four checklists for marine samples, seven pathogen-related sample metadata checklists, a number of project-specific checklists, for example one for patient-derived xenograft models or patient samples and one developed for the Global Microbial Identifier Proficiency Test (https://www.globalmicrobialidentifier.org/workgroups/about-the-gmi-proficiency-tests). The complete list of the ENA checklists including the required fields for each checklist can be viewed and browsed through in the new ENA Browser (https://www.ebi.ac.uk/ena/browser/checklists).

### Data coordination services

We have continued to provide specific data coordination support for our collaborating partners in projects and initiatives across a broad range of scientific areas, expanding our portfolio of collaborations over the last year. Working closely with our partners, we provide support in data sharing, analysis, archiving, search and presentation services through often dedicated search and discovery portal application program interfaces (APIs) and/or graphical user interfaces. This service is extremely valuable to all ENA end users because of its direct link to setting standards and improving the quality and richness of content. The expansion of the analysis types and addition of standards support for metagenomic assembly data described above (see the ‘New Data Types’ and ‘Standards’ sections) have resulted from a data coordination service; this work improves search and discoverability of all assembly types, and in particular metagenomic assemblies that are growing in number. The ENA Rulespace (https://www.ebi.ac.uk/ena/browser/rulespace) is a further example for a service that is developed to provide improved search and synchronization tools for ENA. This service was driven specifically to serve custom views of eukaryote diversity-related content. The Rulespace service enables the creation and management of user-defined rules, and metadata relating to these rules, that can be shared with other interested parties and that are used to define searches on services such as the ENA Discovery API (see also under the ‘ENA Browser’ section). Our current portfolio includes partners from pathogen surveillance and outbreak genomics using the COMPARE data hub system (9) and the Pathogen Portal (https://www.ebi.ac.uk/ena/pathogens/home), livestock functional genomics under the FAANG collaboration (10), metagenomics communities through the Metagenome Exchange (https://www.ebi.ac.uk/ena/registry/metagenome/api/) and MGnify (11) projects, stem cell data through HipSci (12), marine projects such as Tara Oceans (https://www.ebi.ac.uk/ena/about/tara-oceans-assemblies) (13) and Ocean Sampling Day (14) and microbial eukaryote biodiversity projects such as UniEuk (15). A list of our current collaborations and their descriptions can be found at https://www.ebi.ac.uk/ena/browser/about/data_coordination.

### The new ENA Browser

**Figure 3.**
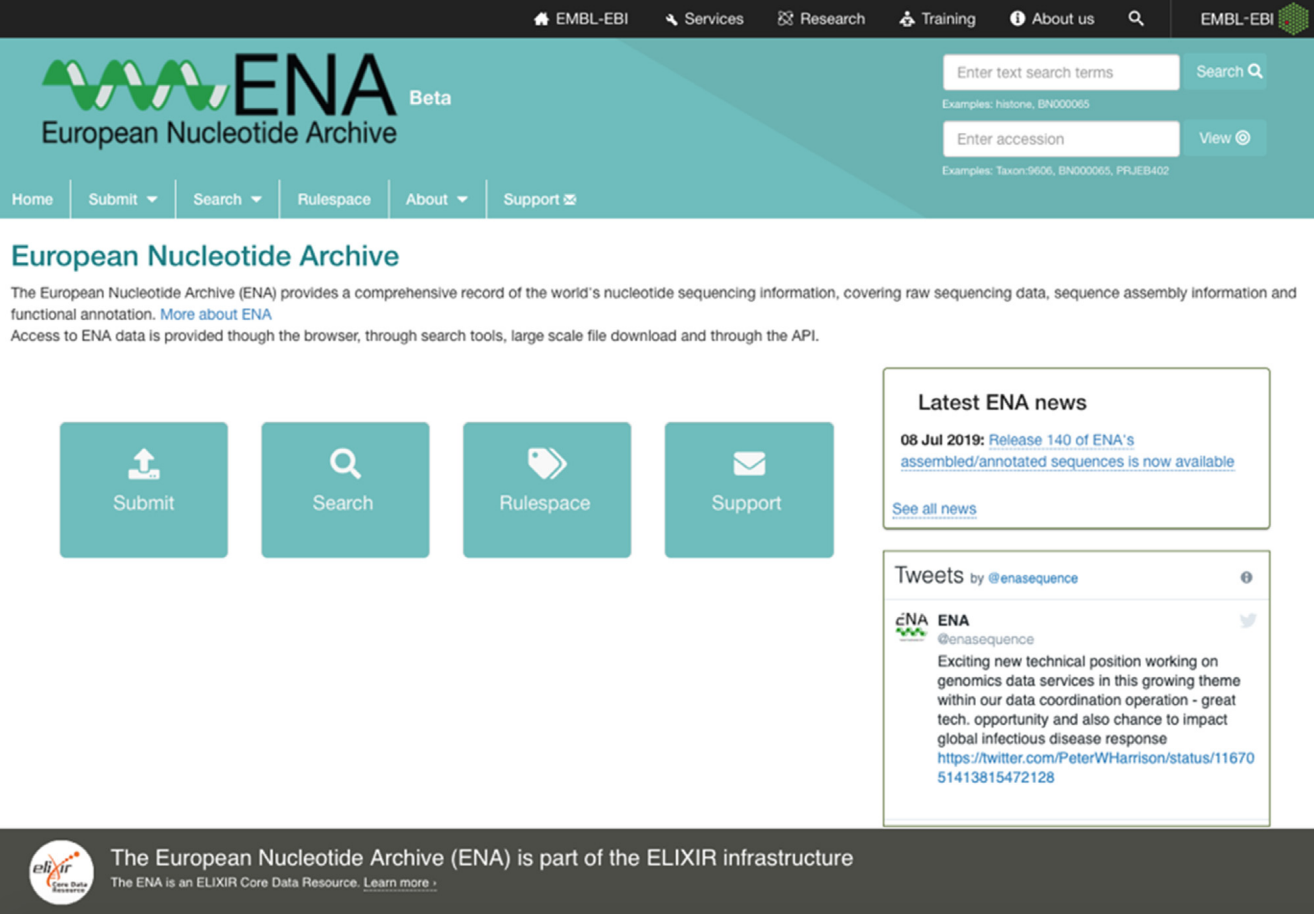
The new ENA Browser, showing its streamlined landing page.

A particular focus for the year has been the development of the new ENA Browser (https://www.ebi.ac.uk/ena/browser/home). This features a completely new modern technology stack (Angular: https://angular.io/; Material: https://material.angular.io/; MongoDB: https://www.mongodb.com/; Vertica: https://www.vertica.com/; Oracle: https://www.oracle.com/; Spring Boot: https://spring.io/projects/spring-boot), a move to microservices for improved maintainability, a complete review and modernization of all previous browser features, a streamlined and simplified user experience and the addition of key new features that improve data discovery and access. The streamlined design focuses each data view on the most important information for the user; this has potential to boost the user experience, make navigation more intuitive and promote easy access to the underlying data. For example, the new homepage features quick access buttons to key site sections, a redesigned tab and page structure, and both a direct accession access and free text search boxes (Figure 3).

**Figure 4.**
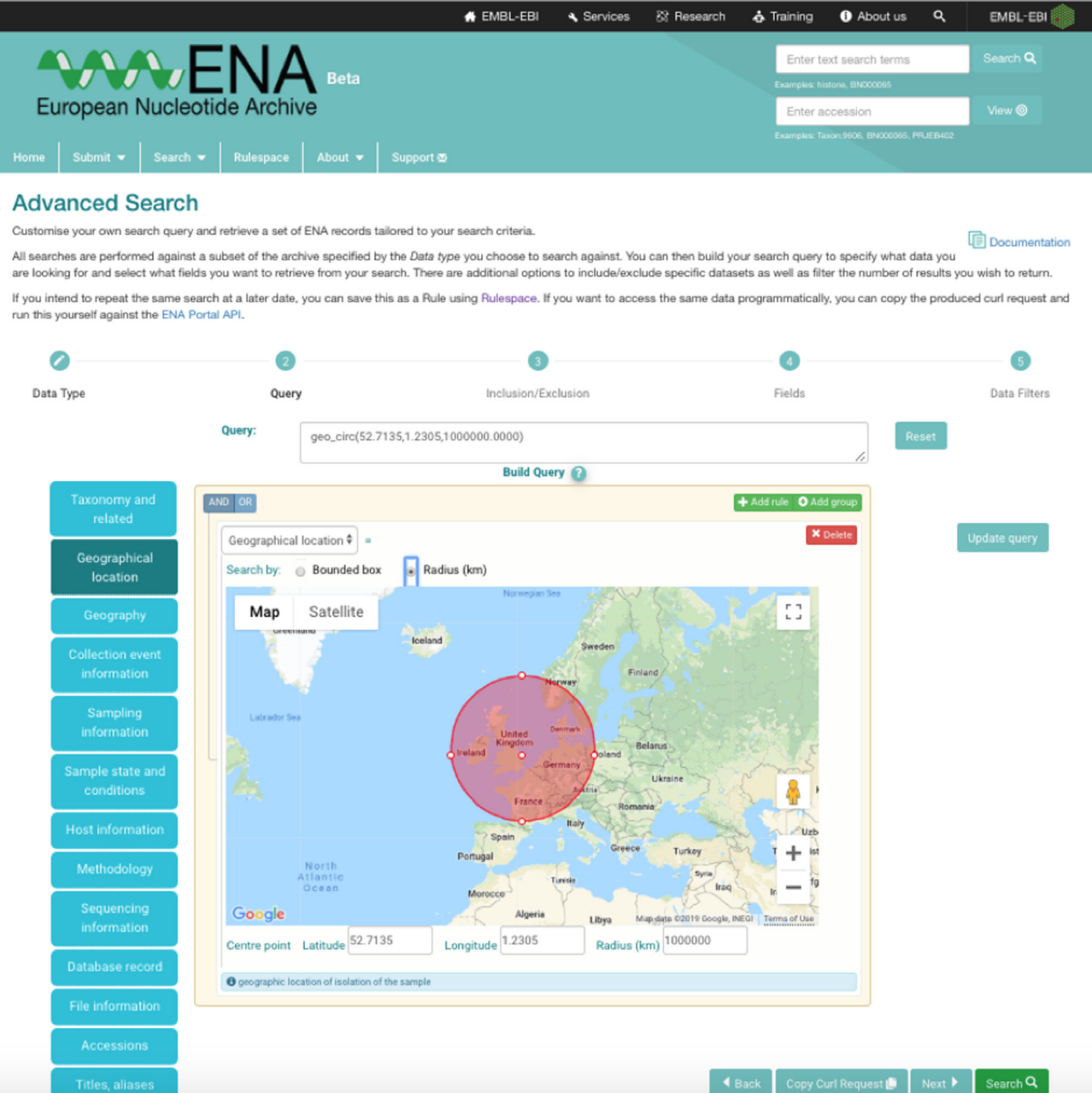
Advanced search query interface for constructing complex searches, for example geographical boundaries.

Search has been overhauled in the new browser with improvements to existing search interfaces and addition of new features. We offer five distinct search interfaces: free text search (simple keyword search), sequence similarity search (BLAST search), sequence version archive search (find non-current sequence versions), cross-reference search (search our extensive array of cross-references and extended annotations from an increasing number of external databases and resources) and a new advanced search service. Advanced search enables the guided construction of complex queries using a range of predefined filters, combined with autocompletion assistance for many fields (Figure 4), with the interface constructing the query language on the user’s behalf. Users can refine the results output using inclusion and exclusion by accession. As the query language is the same as for our API interfaces, the browser features a copy to cURL command button so that a query constructed in the browser can be easily utilized programmatically. CURL is a widely used command line tool for web address-based API interactions, such as those with the ENA APIs, and allows for the transfer of data and files. For example, the following is an example of an advanced search query for all human raw reads in ENA copied to a cURL command to run the same search programmatically, ‘curl -X POST -H “Content-Type: application/x-www-form-urlencoded” -d “result=read_run&query=tax_eq(9606)&format=tsv” https://www.ebi.ac.uk/ena/portal/api/search’.

**Figure 5.**
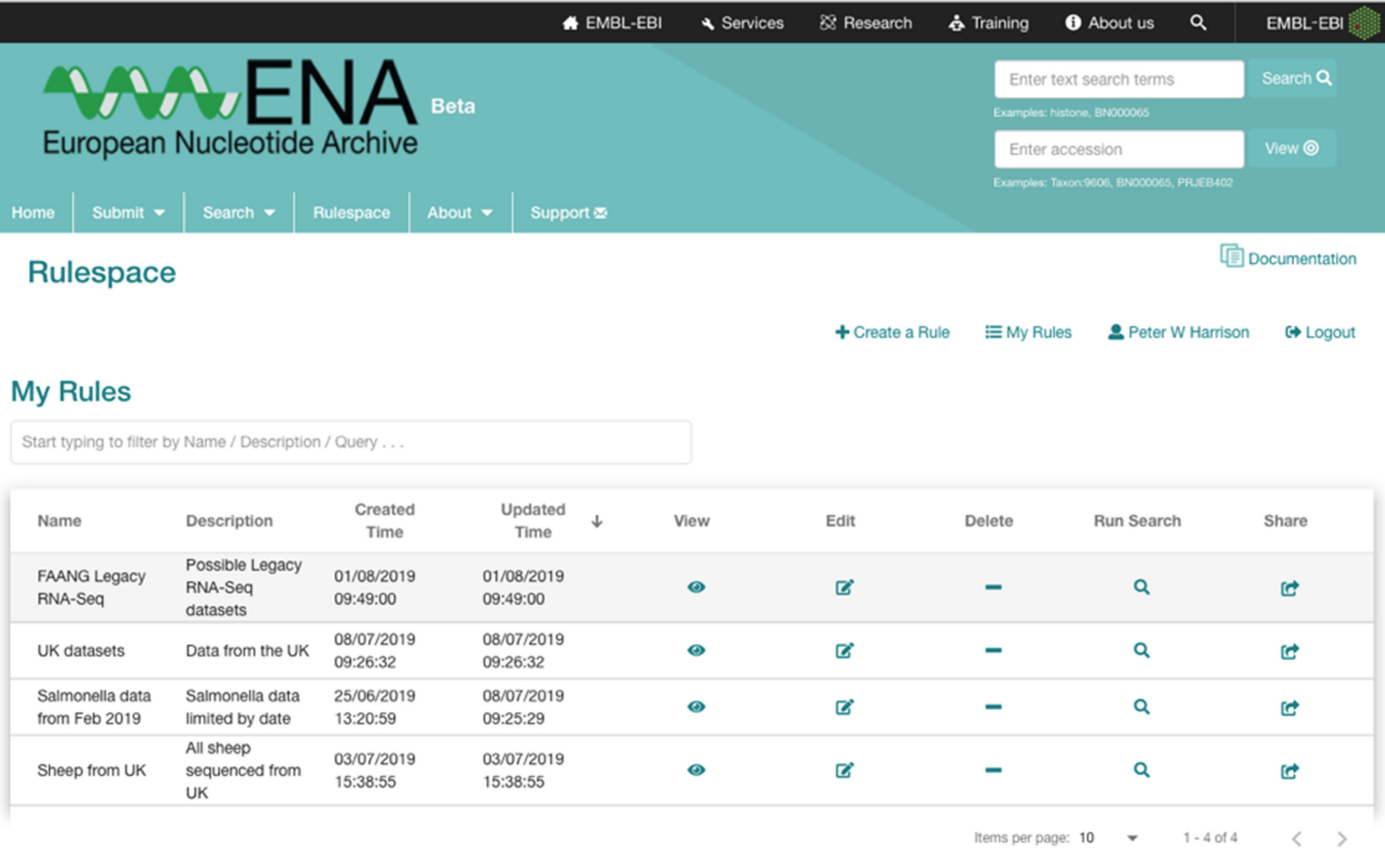
Rulespace interface for managing saved user advanced queries.

Rulespace is a completely new feature of the ENA Browser that allows users to save advanced search queries to their own account, to re-run the same query as new data emerges and also to share the query with collaborators to enable work on identical datasets. An authenticated management interface (Figure 5) enables a user to edit, run or share any previously saved rule queries that each have a user-provided title and description to aid identification. The queries are particularly powerful when the ‘Last updated’ field is included as it allows users to continually return to Rulespace to obtain updated records since a given date. For example, this can be set to be the last time they ran the query. This service is particularly powerful for consortiums and projects that wish to generate and distribute to all of their members a saved custom ENA advanced search. Additionally, Rulespace can also be managed programmatically through its API interface (https://www.ebi.ac.uk/ena/rulespace/api/) that enables creation, management and exploitation of the saved custom queries programmatically.

The new browser sits upon a new public ENA Browser API (https://www.ebi.ac.uk/ena/browser/api/) that was released early in 2019 and serves direct programmatic access. It provides a significant improvement in stability and performance over the previous programmatic data access that was integrated with the old browser and thus subject to file system performance bottlenecks. Browser API is focused on fast sequence retrieval by accession, but works perfectly in tandem with the ENA Portal (Discovery) API (https://www.ebi.ac.uk/ena/portal/api/) that supports powerful search across metadata fields. These APIs work together to provide an integrated search and retrieval programmatic service. Each of the APIs have Swagger interfaces to assist with query construction, configurable outputs and pre-publication authenticated data access. A Swagger user interface helps users easily consume our new APIs, providing easily navigable documentation, a clear overview of the available endpoints and by enabling test queries assists with the design of commands to consume our data and services (https://swagger.io). With the new MongoDB backed deployment of the APIs, we have significant flexibility for future scalability with an easily adaptable database schema design and the option of sharding over an increasing number of machines. This allows us to more easily respond to future changes in metadata, data types and technologies, and to distribute ever more complex queries from an increasing user base over a scalable system.
